# Reduction in Toxicity of Polystyrene Nanoplastics Combined with Phenanthrene through Binding of Jellyfish Mucin with Nanoplastics

**DOI:** 10.3390/nano12091427

**Published:** 2022-04-22

**Authors:** Sun Woo Geum, Min-Kyeong Yeo

**Affiliations:** 1 Department of Applied Environmental Science, Graduate School of Engineering, Kyung Hee University, 1732 Deogyeong-daero, Giheung-gu, Yongin-si 17104, Gyeonggi-do, Korea; tjsdn3486@khu.ac.kr; 2 Department of Environmental Science and Engineering, College of Engineering, Kyung Hee University, 1732 Deogyeong-daero, Giheung-gu, Yongin-si 17104, Gyeonggi-do, Korea

**Keywords:** polystyrene nanoplastics, jellyfish mucin, reduce toxicity

## Abstract

Mucin (Mu), a biological substance extracted from jellyfish (*Aurelia aurita*), was used to reduce the toxic effect of polystyrene nanoplastics (PS-NP) combined with phenanthrene (Phe) in the aquatic environment of zebrafish (*Danio rerio*), among other aquatic organisms. Mu showed a high binding capacity, as it bound to 92.84% and 92.87% of the PS-NPs (concentration of 2.0 mg/L) after 0.5 h and 8 h, respectively. A zebrafish embryo development test was conducted to check for any reduction in toxicity by Mu. When exposed to PS-NP + Mu and PS-NP + Phe + Mu, respectively, the hatching rates were 88.33 ± 20.21% and 93.33 ± 2.89%, respectively; these results were not significantly different from those of the control group. However, the hatching rate with the addition of Mu increased, compared to that of the PS-NP (71.83 ± 13.36%) and Phe (37.50 ± 19.83%) treatments, and the morphological abnormality rate decreased. The presence of Mu was also found to obstruct the absorption of PS-NP and PS-NP + Phe by the zebrafish. When zebrafish embryos were exposed to PS-NP at a concentration of 5.0 mg/L, the hatching rate differed significantly from that of the control group, and the expression of CAT and p53 genes increased significantly, but the expression of Bcl-2 decreased significantly. An mRNA sequence analysis revealed that the gene expression levels of the test group containing Mu were similar to those of the control group. These results infer that Mu can be used as a biological material to collect and remove PS-NPs from aquatic environments and reduce toxicity.

## 1. Introduction

Polystyrene (PS) is a plastic material used in producing styrofoam, which is used in food containers and packaging products [1]; it is one of the most abundant microplastics found in the marine environment [2]. Plastic waste discharged into the environment does not decompose rapidly, but goes through a decomposition process, involving photolysis, oxidation, abrasion, hydrolysis, and biodegradation over a long period [3,4,5], and plastics < 100 nm are referred to as “nanoplastics (NP)” [6,7,8,9].

Polystyrene nanoplastics (PS-NP) produce reactive oxygen species (ROS), which ultimately result in oxidative stress [10,11,12,13]. Catalase (CAT), superoxide dismutase (SOD), and glutathione s-transferase (GST) are antioxidant enzymes that form the first line of defense against oxidative stress, by removing ROS [14,15]. The impairment of these enzymes causes damage to the cell membrane and DNA, resulting in a loss of defense capability and cell damage [15]. Zebrafish larvae that were exposed to PS-NP showed a decreased expression of the oxidative stress-related gene CAT [16]. Additionally, the exposure of PS-NP to the fish cell line SaB-1 affects the transcription of Bcl-2 and CAT genes [17]. Amine-modified PS-NP causes cell toxicity by caspase-mediated apoptosis at relatively low concentrations [18], due to cell membrane destruction [18,19]. 

Due to their hydrophobicity and small size, NPs have a large surface area-to-volume ratio, making them efficient adsorbents for toxic chemicals, such as the heavy metals and polycyclic aromatic hydrocarbons (PAHs) contained in hydrophobic organic contaminants (HOCs) [20,21,22,23]; NPs can affect aquatic species or accumulate through the food chain, by adsorption and desorption [23]. Phenanthrene (Phe) is a low-molecular-weight PAH, with higher bioavailability than high-molecular-weight PAHs; Phe is more water soluble and more likely to bioaccumulate in aquatic organisms [24]. The PAHs that are discharged into wastewater adversely affect aquatic organisms [25], causing oxidative stress, immunotoxicity, carcinogenicity, and developmental toxicity [26,27,28,29,30,31]. Phe is one of the most abundant PAHs in the atmosphere [32], and large amounts are introduced into the aquatic environment through oil spills from ships. Although its impact on aquatic ecosystems requires urgent investigation, few studies have researched the effect of its adsorption with NPs in the aquatic ecosystem.

NPs enter aquatic environments through the wastewater treatment process and are either discharged into lakes or rivers, or remain in sludge [33]. Highly efficient physicochemical treatment is generally used to treat wastewater, which further undergoes post-treatment, due to secondary contamination by solid waste or sludge [34]. It is, therefore, necessary to develop and use biological treatments that do not cause secondary contamination.

Among the biological materials used to collect nanomaterials, hydra (*Hydra magnipapillata*) protein and antimicrobial peptides (AMPs) have proved to be effective in trapping silver nanoparticles and reducing their toxicity [35,36]. Jellyfish extract at immunity reaction (JEI) of jellyfish (*Aurelia aurita*), which also belongs to the phylum cnidaria, effectively collects silver nanoparticles and reduces nanotoxicity [37,38]. However, as JEI can only be extracted from live jellyfish, which are only abundant during a certain period, there were challenges in the supply and storage of the substance.

A mucin-based glycoprotein of jellyfish, qniumucin [39], can be considered an alternative to solving this limitation. Qniumucin has almost no peptide sequences, except for the mucin (Mu) part; thus, it should be non-toxic to living organisms and only cause a mild biological reaction, in the form of an allergy [40]. Because of its non-toxicity and availability in all parts of the jellyfish, the jellyfish Mu is expected to significantly reduce the risks associated with mass production and supply [40,41]. Currently, jellyfish Mu has only been studied as a substitute for human Mu for some medical purposes [42], and the possibility of using this for the biological treatment of wastewater, to avoid secondary contamination, has not been explored.

This study proposes an improved extraction and storage method for the glycoprotein Mu, which is the main component of JEI. The applicability of the Mu on biological treatment (collecting PS-NPs) and its ability to bind to PS-NPs was quantitatively evaluated through a fluorescence spectrophotometer. Additionally, PS-NP, Phe, and PS-NP + Phe were exposed to the zebrafish embryos, and the effects of Mu (toxicity reduction) were tested, by comparing it with the binding groups (PS-NP + Mu, Phe + Mu, PS-NP + Phe + Mu). Furthermore, the effect of exposure in each test group on the genes was analyzed using mRNA sequencing, and the expression of SOD1, CAT, GPx1a, Bax, Bcl-2, p53, and caspase-3 (genes related to oxidative stress and apoptosis) was analyzed, with respect to the toxicity associated with PS-NP and Phe.

## 2. Materials and Methods

### 2.1. Jellyfish Mu Extraction

The jellyfish (*Aurelia aurita*) from the South Sea of Korea were used in this study. They were provided by the Korea Jellyfish Lab (Seoul, Korea), and all the organisms were stored at −80 °C before use.

The extraction method in reference [39] was modified for Mu extraction. Immediately before extraction, the jellyfish were thawed at room temperature and washed 3 times with tertiary distilled water to remove the salt. They were then shredded and finely broken down for 1 min, using an ultrasonic probe processor (Vibra-Cell, Sonics, Newton, CT, USA). This was followed by the addition of 0.2% NaCl twice the volume of the broken-down jellyfish samples, and the mixture was stirred for 24 h at 4 °C. After centrifugation at 10,000× *g* for 10 min at 4 °C, ethyl alcohol (~99.9% pure) thrice the volume of the supernatant was added and maintained overnight at 4 °C. The resulting gel-like precipitate was centrifuged again at 10,000× *g* for 10 min at 4 °C, and the resulting pellet was collected. The pellet was again centrifuged at 10,000× *g* for 10 min at 4 °C to remove the ethyl alcohol, and left at room temperature (20 ± 5 °C) for 20 min, before washing three times with tertiary distilled water. After drying, the pellet was stored at −80 °C until use.

### 2.2. Physicochemical Properties of PS-NP, PS-NP + Phe, and Mu Mixtures

A plain PS-NP suspension at a concentration of 2.7% (*w*/*v*) and a fluorescent PS-NP suspension at a concentration of 2.6% (*w*/*v*), both with particle sizes of 50 nm, were purchased from Polysciences (Warrington, PA, USA). Plain PS-NP was used for assessing the physicochemical properties and conducting the toxicity tests, while fluorescent PS-NP was used for the binding capacity experiments and observations involving a fluorescence microscope. Phe (~98% pure) and dimethyl sulfoxide (DMSO, ~99.5% pure), purchased from Sigma-Aldrich (St. Louis, MO, USA), were used. The solution for the Phe exposure test was prepared by diluting with 0.1% (*v*/*v*) DMSO, as specified in ISO 7346-3 for the standard dilution water (ISO-water) [43].

The surface morphology of PS-NP, after binding to Phe and Mu, was analyzed using field emission scanning electron microscopy (FE-SEM) (LEO SUPRA 55, Carl Zeiss, Oberkochen, Germany; 10 kV). Additionally, particle size (ELS-Z2, Otsuka Electronics, Tokyo, Japan) and zeta potential (ELSZ-2000ZS, Otsuka Electronics) analyses were conducted to estimate the changes in the surface charge and particle size.

The final concentrations prepared for the experiments were as follows: PS-NP: 5.0 mg/L, Phe: 1.0 mg/L, and Mu: 50 μg/mL. Tertiary distilled water was added to these solutions, and an ultrasonic processer was used for periods of 5 min, 30 s, and 3 min to disperse the PS-NP, Phe, and Mu, respectively, immediately before the experiment. PS-NP + Mu and PS-NP + Phe + Mu were homogenized using a vortex mixer, where the PS-NP or PS-NP + Phe sample was mixed with the Mu sample at a volume ratio of 1:1. PS-NP + Phe was prepared one day prior to conducting the experiment because of the amount of time required for Phe to be adsorbed by the PS-NP [44,45]. Each substance was mixed with tertiary distilled water, dispersed using an ultrasonic processor for 5 min, and then mixed again at 55 rpm in a rocker (Compact ROCKER CR95, FINEPCR, Gunpo, Korea) for 24 h in a dark room at 20 °C. The solution was then measured after dispersing for 30 s, using an ultrasonic processor immediately prior to conducting the experiment. 

### 2.3. Test Organism and Exposure Conditions

The zebrafish (*Danio rerio*, wild type) used in this study were bred in our laboratory and were 6 to 7 months old. They were managed according to the method in references [37,46]. The water temperature was maintained at 28.5 ± 1 °C, with a photoperiod of 14/10 h, electrical conductivity of 211.4 ± 1.2 μS/cm, pH of 7.02 ± 0.5, chlorine concentration of 0.8 mg/L, and total organic carbon of 0.27 mg/L. Dried flakes (TetraMin, Melle, Germany) and brine shrimp (*Artemia* sp.) were fed to the organisms twice a day. To maintain a natural aquatic environment and minimize the effect of chlorine on the zebrafish embryos, they were washed twice in 24 h with dechlorinated tap water (20.5 ± 1 °C). The washed embryos were placed in a 6-well plate, with 20 embryos per well.

To minimize the effect of outside plastic, glassware was used for all experimental purposes. To maintain the same embryonic stage in all the experimental groups, they were simultaneously exposed at 1 h post-fertilization, and were maintained at 28.5 ± 1 °C during the embryonic development. The embryonic development stage was observed using a stereomicroscope system (SZ61, Olympus, Shinjuku, Japan) after 0, 4, 8, 12, 24, 32, 48 and 72 h exposure, according to the method in references [38,47]. Dead embryos were removed immediately to avoid contamination of the test solution. The number of hatched zebrafish larvae was counted after 72 h of exposure to measure the hatching rate and abnormal rate. The embryos that did not hatch after 72 h were considered dead. All experiments were repeated 3 times.

The Mu stored at −80 °C was placed in dechlorinated water for 24 h just before exposure, and dispersed for 3 min using an ultrasonic processor. A pre-test for exposure was performed with 1, 5, 10, and 50 μg/mL of Mu to select the optimal concentration, among which 50 μg/mL was selected. 

Mu-bearing and Mu-absent PS-NP, Phe, and PS-NP + Phe were placed in dechlorinated water for 24 h and then dispersed using an ultrasonic processor, with dispersion conditions identical to the experiments for estimating the physicochemical properties. The experimental groups were PS-NP, Phe, PS-NP + Phe, Mu, PS-NP + Mu, Phe + Mu, and PS-NP + Phe + Mu, whose concentrations were selected through the pre-test (PS-NP = 5.0 mg/L, Phe = 1.0 mg/L, and Mu = 50 μg/mL).

### 2.4. Quantitative Analysis of the Binding Capacity of Jellyfish Mu and PS-NP

Fluorescence detection was used to quantify the PS-NPs, and all PS-NP-bearing samples were prepared using fluorescent PS-NP for binding capacity evaluation. A standard solution of fluorescent PS-NP was prepared and fluorescence was detected, using a fluorescence spectrophotometer (FL6500 Luminescence system, PerkinElmer Inc., Waltham, MA, USA). The concentrations of the fluorescent PS-NP standard solutions were 0.75, 1.00, 1.25, and 2.00 mg/L, and they were prepared immediately prior to the experiment, by mixing fluorescent PS-NP with tertiary distilled water and then dispersion for 5 min in an ultrasonic processor. The excitation wavelength was 441 nm, and the emission wavelength was 450–800 nm. Figure S1 shows the standard curve with the concentration (mg/L) of the standard solution on the *x*-axis and the fluorescence intensity on the *y*-axis. It is based on the fluorescence value measured at excitation wavelength 441 nm and emission wavelength 485 nm, in accordance with the datasheet of Polysciences. 

To evaluate the binding capacity of the jellyfish Mu with PS-NP, both substances were mixed separately with tertiary distilled water immediately before the experiment, followed by dispersion of 3 min and 5 min, respectively. During the test, 2 mg/L of PS-NP and 100 μg/mL of Mu were mixed at a volume ratio of 1:1. The time of mixing was set as 0, and samples were collected at 0.5, 1, 2, 4, and 8 h. Each sample separated over time into the supernatant and precipitate; the supernatant was regarded as the PS-NP not bound to Mu and was used to evaluate the binding capacity. The fluorescence detection conditions were identical to those of the PS-NP standard solution, and the fluorescence value of PS-NP not bound to Mu was substituted into the standard curve and converted into concentration.

### 2.5. Analysis of Substances Penetrated and Absorbed by the Zebrafish Embryos 

Different concentrations of Phe (0.1, 0.5 and 1.0 mg/L) were pre-tested before exposure to the zebrafish embryo to find the optimum value. These concentrations were combined with 5.0 mg/L of PS-NP and their absorption by zebrafish embryos was analyzed using fluorescent PS-NP. Additionally, to examine the absorption of Mu-bound PS-NP by the zebrafish embryos, they were studied under a fluorescence microscope. Three embryos and larvae after 24 and 72 h exposure were randomly selected from each of the exposure groups, they were washed thrice within 24 h in dechlorinated water, anesthetized with 0.16% tricaine (3-aminobenzoic acid ethylester), and embedded onto methylcellulose, before being observed through a fluorescence microscope (IX73, Olympus). The fluorescence intensity of the image was calculated using the ImageJ (Wayne Rasband, National Institutes of Health, Bethesda, MD, USA) software.

The concentration of Phe selected from the pre-test was 1.0 mg/L, and the observed experimental groups were PS-NP, PS-NP + Phe, PS-NP + Mu, and PS-NP + Phe + Mu. All exposure conditions were identical to those of the pre-test for exposure.

### 2.6. Gene Analysis

#### 2.6.1. mRNA Sequence Analysis

The total RNA was extracted using the TRIzol Reagent (Invitrogen, Waltham, MA, USA). RNA quality was measured using an Agilent 2100 bioanalyzer (Agilent Technologies, Amstelveen, The Netherlands), and the RNA was quantified using an ND-2000 Spectrophotometer (Thermo Fisher Scientific, Inc., Waltham, MA, DE, USA).

The gene library was prepared from the total RNA using an NEBNext Ultra II Directional RNA-Seq Kit (New England BioLabs, Inc., Ipswich, MA, USA). The mRNA was extracted using a Poly (A) RNA Selection Kit (LEXOGEN, Inc., Vienna, Austria). cDNA was synthesized and sheared from the extracted mRNA, according to the method presented in the kit. Illumina Index Primers 1–12 were used for indexing, and polymerase chain reaction (PCR) in the amplification step. An Agilent 2100 Bioanalyzer (DNA High Sensitivity Kit, Agilent Technologies, Inc., Santa Clara, CA, USA) was used to evaluate the mean fragment size and confirm the library. The library was quantified using a StepOne Real-Time PCR System (Life Technologies, Inc., Carlsbad, CA, USA); high-throughput and paired-end 100 bp sequencing was performed using a NovaSeq 6000 (Illumina, Inc., San Diego, CA, USA).

#### 2.6.2. Data Analysis

The quality control of the raw data from the mRNA sequencing was performed by FastQC (https://www.bioinformatics.babraham.ac.uk/projects/fastqc, accessed on 20 March 2022). After removing the adapter and low-quality reads (<Q20) through FASTX_Trimmer (http://hannonlab.cshl.edu/fastx_toolkit, accessed on 1 December 2021) and BBMap (https://sourceforge.net/projects/bbmap, accessed on 1 December 2021), the trimmed reads were mapped to the reference genome for zebrafish (danRer10) and registered in the UCSC Genome Browser, using TopHat [48]. Normalized fragments per kb per million reads (FPKM), based on the quantile normalization method that uses EdgeR within R [49], was used to calculate the gene expression levels. Data mining and visualization were performed using ExDEGA v. 3.2.1 (Ebiogen Inc., Seoul, Korea). The differentially expressed genes were analyzed through upregulated or downregulated expressed values, by comparing the log2 transformed normalized data of each FPKM value of the experimental group with that of the control group. The expression level of the gene was used at a fold change of ≥2 or ≤0.5, and *p* < 0.05. For the clustering heatmap of the expressed genes, z-score values were calculated by subtracting the mean value from the log10 transformed normalized data and dividing by the standard deviation.

#### 2.6.3. Quantitative Analysis of Gene Expression

RNA was extracted for quantitative PCR (qPCR) analysis, according to the instructions of the Qiagen RNeasy Mini Kit (Qiagen, Hilden, Germany), whereby extraction was performed from the entire body of the zebrafish larvae that hatched after 72 h of exposure. For each experimental group, the collected larvae were transferred to a 1.5 mL tube, where the exposed material was washed thrice with phosphate-buffered saline (Welgene, Gyeongsan, Korea) and then homogenized with a RLT buffer (350 μL.), using a homogenizer (BioMasher-II, NIPPI, Tokyo, Japan). After centrifugation at 13,000× *g* and 20 °C for 3 min, 350 μL of the supernatant was collected and mixed with 350 μL of 70% ethyl alcohol. The 700 μL mixture was transferred to a RNeasy spin column, where it was centrifuged at 10,000× *g* and 4 °C for 15 s, and washed once with a RW1 buffer (700 μL) and twice with a RPE buffer (500 μL). The RNease-free water (30 μL) was dropped into the center of the spin column and centrifuged at 10,000× *g* and 4 °C for 1 min to extract the RNA. After measuring the concentration and purity of the RNA using a NanoPhotometer (N60, IMPLEN, Munchen, Germany), cDNA was synthesized using a FIREScript^®^ RT cDNA synthesis MIX (Solis Biodyne, Tartu, Estonia). The synthesized cDNA was stored at −20 °C before the analysis of qPCR.

qPCR was performed on a LineGene 9600 (BIOER, Hangzhou, China), using a 5x FIREPol EvaGreen qPCR Superix (Solis Biodyne). A cycle consisting of initial denaturation at 95 °C for 10 min, DNA denaturation at 95 °C for 30 s, primer annealing at 60 °C for 20 s, and elongation at 72 °C for 30 s, was repeated 40 times (cycles). Table 1 shows the primer used in the qPCR analysis. The gene expression level was normalized by the housekeeping gene β-actin, and calculated using the 2^−ΔΔCt^ method. The melting curve of the amplified product was examined to confirm that qPCR was properly performed.

### 2.7. Statistical Analysis

Statistical analysis was performed using the Statistical Package for the Special Science (v. 23.0) of IBM. A one-way analysis of variance was performed to compare the hatching rate, abnormality rate, gene expression, and binding capacity of the zebrafish embryo toxicity test. The results were obtained using Turkey’s post-hoc test for multiple comparisons. All experiments were repeated thrice, and the differences were considered statistically significant at *p* < 0.05 and *p* < 0.01. 

## 3. Experimental Results and Discussion

### 3.1. Physicochemical Properties of PS-NP, Phe, and Mu Mixtures

The PS-NP was sphere-shaped, with a diameter of 50 nm (Figure S2a), and its size and shape was the same in PS-NP + Phe (Figure S2b). Mu did not exhibit a specific form, and some Mu clustered to form agglomerates (Figure S2c). The PS-NP was adsorbed to Mu to form PS-NP + Mu agglomerates (Figure S2d). Furthermore, a mixture of PS-NP + Phe and Mu formed PS-NP + Phe + Mu agglomerates, which were more widely spread than the PS-NP + Mu agglomerates (Figure S2e).

The particle size results showed that the sizes of the PS-NP particles dispersed in tertiary distilled water increased from 50 nm to 2132 ± 90.86 nm and that of PS-NP + Phe increased to 1614 ± 85.56 nm (Table S1). The particle sizes of the PS-NP bonded onto Mu that formed agglomerates increased by 1.59 times, compared to those of the PS-NP particles, and those of PS-NP + Phe + Mu increased by 4.51 times, compared to those of PS-NP +Phe (7277 ± 277.3 nm) (Table S1).

The further increase in the PS-NP particle size (Table S1) likely occurred because certain nanoparticles cannot maintain stable dispersion conditions in water without a dispersant (such as citrate) or surface coating (such as polyethylene glycol or polyvinylpyrrolidone) [50]. Particularly, nanoparticles that have not undergone any special treatment, such as dispersion or surface coating, may form agglomerates when discharged to the environment, thereby increasing the hydrodynamic diameter [51,52]. PS-NP can bind to proteins, which have been reported to bind to the surface of particles that determine the biological identity of nanoparticles, commonly referred to as protein corona [53]. Therefore, it is presumed that Mu, a type of glycoprotein extracted from jellyfish, can bind to PS-NPs dispersed in water. Additionally, charged or hydrophobic nanoparticles tend to bind better with proteins and have more stable interactions than the hydrophilic nanoparticles [53]. In the case of PS-NP + Phe + Mu, it was observed that the particle size increased significantly than that of the PS-NP, presumably because PS-NP + Phe was more hydrophobic than the PS-NP, binding more stably with Mu.

The zeta potential of the PS-NP, which denotes the section where the stability between particles decreases and the attractive forces between them start to have a bigger impact than repulsive forces, was −7.49 ± 1.12 mV [54]. These results are similar to those of the particle size analysis, where dispersion conditions cannot be maintained in water without the dispersant or surface coating, and particles form agglomerates (Table S1). The zeta potential for PS-NP + Phe was −12.34 ± 1.95 mV, which is more stable than the PS-NP, and forms fewer agglomerates. This was also consistent with the particle size analysis results, where the particle size of PS-NP + Phe was smaller than the PS-NP (Table S1). The zeta potentials for PS-NP + Mu and PS-NP + Phe + Mu were −3.37 ± 0.58 and −5.13 ± 0.07 mV, respectively, which suggests that binding with Mu helped to maintain more stable dispersion (Table S1). Furthermore, the zeta potential of PS-NP + Phe + Mu was less than that of PS-NP + Mu, and this was probably because the more stable interaction enabled easier binding with Mu.

### 3.2. Evaluation of the Binding Capacity of Jellyfish Mu and PS-NP

After 0.5 h, 7.16% (0.14 ± 0.04 mg/L) of the injected PS-NP was detected in the supernatant, indicating that the binding capacity of the injected PS-NP with Mu was 92.84% (1.86 ± 0.04 mg/L). After 1 h, the binding capacity was observed to decrease slightly to 90.40% (1.81 ± 0.01 mg/L), probably because when secreted from the body, Mu absorbs water and expands. The slight decline in the binding capacity occurred because some PS-NPs were desorbed while adsorbing water when Mu was expanding, but the extent of desorption was insignificant. After 4 h, the binding capacity again increased to 91.39% (1.83 ± 0.03 mg/L), and after 8 h, it recovered to 92.87% (1.86 ± 0.01 mg/L) (Table 2). Statistically, the PS-NP concentration was significantly reduced after 0.5 h compared to the concentration at 0 h, and the same trend was observed after 1, 2, 4, and 8 h (*p* < 0.01). Thus, it was confirmed that the binding capacity was continuously maintained even after 8 h (Table 2).

### 3.3. Analysis of Substances Penetrated and Absorbed by the Zebrafish Embryos

#### 3.3.1. Effects of PS-NP, Phe and PS-NP + Phe without Mu

**Figure 1:**
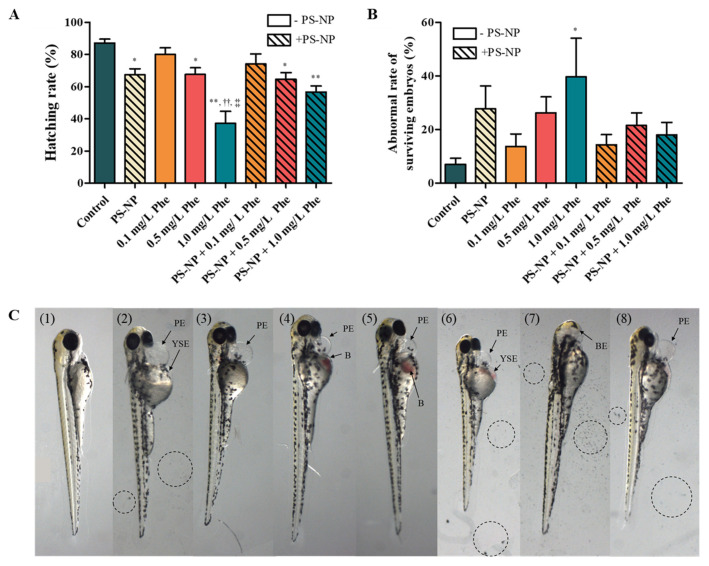
(**A**) Hatching rate and (**B**) abnormal rate of surviving embryo of zebrafish exposed to PS-NP (5.0 mg/L), Phe (0.1, 0.5, and 1.0 mg/L), and PS-NP (5.0 mg/L) + Phe (0.1, 0.5, and 1.0 mg/L). *: control vs. experimental group; (*p* < 0.05), **: control vs. experimental group; (*p* < 0.01), ^††^: 0.1 mg/L Phe vs. 1.0 mg/L Phe; (*p* < 0.01), ^‡‡^: 0.5 mg/L Phe vs. 1.0 mg/L Phe; (*p* < 0.01). (**C**) Zebrafish larva exposed to (**1**) control, (**2**) PS-NP (5.0 mg/L), (**3**) 0.1 mg/L Phe, (**4**) 0.5 mg/L Phe, (**5**) 1.0 mg/L Phe, (**6**) PS-NP + 0.1 mg/L Phe, (**7**) PS-NP + 0.5 mg/L Phe, and (**8**) PS-NP + 1.0 mg/L Phe (PE, pericardial edema; YSE, yolk-sac edema; B, blood; BE, bilateral eyelessness) (The circles in (**2**,**6**–**8**) represent PS-NP).

The hatching rate of the experimental group exposed to PS-NP was 67.33 ± 11.69%, which was significantly less compared to that of the control group (87.17 ± 8.37%; *p <* 0.05) (Figure 1A). In the experimental group, where Phe was exposed in concentrations of 0.1, 0.5, and 1.0 mg/L, the toxicity increased with the increase in concentration (Figure 1A,B), and at the concentration of 1.0 mg/L, the abnormality rate increased significantly by 5.60 times (*p <* 0.05) (Figure 1B).

The hatching rates of the experimental group exposed to PS-NP + 0.5 mg/L Phe and PS-NP + 1.0 mg/L Phe were slightly lower than that of the experimental group exposed to only PS-NP, but the difference was not statistically significant (Figure 1A). As for the PS-NP + Phe experimental group, the toxicity increased according to the concentration of Phe, but no statistical difference was observed in the hatching and abnormality rates, compared to the experimental group exposed to Phe alone (Figure 1A,B). These results suggest that, due to the adsorption of the PS-NP and Phe, the simultaneous exposure of the PS-NP and Phe on the zebrafish embryos did not increase toxicity.

These results are consistent with recent studies, where when the zebrafish embryos were exposed to 20 nm PS-NP together with Phe, the lethal concentration was slightly lower, compared to when exposed to Phe alone (lethal concentration 50%, LC_50_), but the difference was statistically insignificant [55]. The simultaneous exposure of PS-NP, with a mixture of 36 PAHs contained in the sediments of the river substratum, interfered with PAH absorption and was shown to reduce teratogenicity of the zebrafish embryos [56]. When white seabass (*Atractoscion nobilis*) were exposed to PS-NP and benzo(a)pyrene (BaP) 45 d after hatching, no significant difference was observed in these, compared to the control group [57]. In addition, 20 and 200 μg/L of microplastics (MP) increased the accumulation of Phe in marine medaka (*Oryzias melastigma*) larvae, but no significant biological effect was observed [58].

PS has a relatively higher adsorption capacity than other plastic materials, in particular polyethylene (PE). This is because PE can only generate non-specific van der Waals interaction, whereas the adsorption between PS and HOCs is driven by hydrophobicity, π-π interaction, and the planarity of molecules [59]. Aromatic compound PAHs have planar structures, in which several benzene rings are bonded, and due to this structural characteristic, it creates a possibility of π-cloud overlap with PS, which is the same kind of aromatic compound, and allows closer access to the adsorption surface [60,61]. Additionally, the structure of PS has a wide interlayer spacing between monomers compared to other plastic materials, and these pores or interlayer spacings existing at the molecular level allow planar molecules to penetrate and bond more easily [60]. In fact, since PAH molecules, such as Phe, are planar, adsorption can occur more easily than in polychlorinated biphenyl (PCB), which is a non-planar molecule with the same hydrophobicity [62,63,64,65]. The strong π-π interaction, due to the structural characteristics of PS-NP and Phe, provides a stronger binding, but can induce a very slow desorption process, thereby reducing the bioavailability of Phe in the absorbed organism [66], which presumably decreases the toxicity of Phe. 

Complex morphological abnormalities, such as pericardial edema and yolk-sac edema, were found in the larvae exposed to PS-NP and Phe, and pericardial edema was commonly found in almost all experimental groups (Figure 1C). Table 3 shows the overall morphological abnormalities of the hatched larvae. 

The main cause for the developmental disorders in Phe-exposed fish is Phe-induced cardiac dysfunction, which results in secondary morphological abnormalities that interfere with the development of cardiac morphology [67]. In this study, cardiac anomalies were also observed in the experimental group exposed to Phe, and abnormalities, such as vascular abnormalities, bleeding, yolk edema, and curved spine (Table 3), were presumed to have been derived from the cardiovascular abnormalities caused by Phe [67]. Additionally, immunotoxicity is one of the typical toxic reactions induced by PS-NP and Phe [24,68]. Inflammatory reactions were observed in both the experimental groups exposed to PS-NP and Phe, which were more dose-dependent in the case of Phe (Table 3); this is presumably derived from the immunotoxicity caused by the PS-NP and Phe.

**Figure 2:**
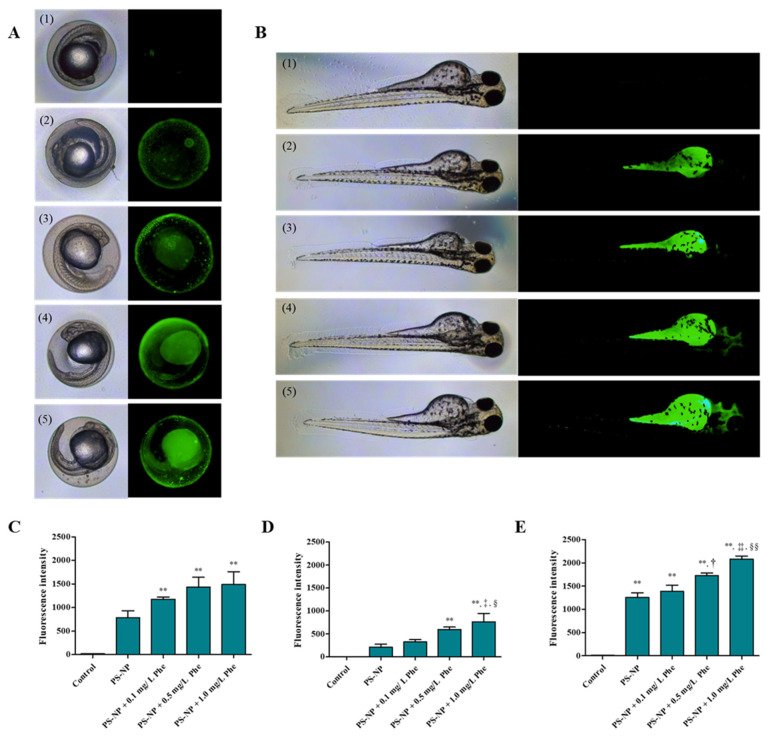
(**A**) Accumulation of PS-NP (5.0 mg/L) in zebrafish embryos according to different Phe concentrations and (**B**) larva. (**1**) Control, (**2**) PS-NP, (**3**) PS-NP + 0.1 mg/L Phe, (**4**) PS-NP + 0.5 mg/L Phe, and (**5**) PS-NP + 1.0 mg/L Phe. (**C**) The quantitative analysis of the fluorescence intensity of the zebrafish embryo 24 h after exposure, (**D**) yolk of the zebrafish embryo 24 h after exposure, and (**E**) zebrafish larva 72 h after exposure (h). **: control vs. experimental group; (*p* < 0.01), ^†^: PS-NP vs. PS-NP + 0.5 mg/L Phe; (*p* < 0.05), ^‡^: PS-NP vs. PS-NP + 1.0 mg/L Phe; (*p* < 0.05), ^‡‡^: PS-NP vs. PS-NP + 1.0 mg/L Phe; (*p* < 0.01), ^§^: PS-NP + 0.1 mg/L Phe vs. PS-NP + 1.0 mg/L Phe; (*p* < 0.05), ^§§^: PS-NP + 0.1 mg/L Phe vs. PS-NP + 1.0 mg/L Phe; (*p* < 0.01).

In the experimental group that was exposed to only the fluorescent PS-NP, fluorescence was mainly detected around the chorion (Figure 2A), and in the PS-NP + Phe experimental group, more fluorescence was detected even in the inside of the chorion, with the increase in the Phe concentration (Figure 2A). The fluorescence intensity of the yolk of the embryo prior to hatching in the experimental groups exposed to PS-NP + 0.1 mg/L Phe, PS-NP + 0.5 mg/L Phe, and PS-NP + 1.0 mg/L Phe increased by 1.54, 2.85, and 3.65 times, respectively, compared to that in the PS-NP, and this increase was statistically significant in the PS-NP + 0.5 mg/L Phe and PS-NP + 1.0 mg/L Phe groups, compared to the control group (*p* < 0.01) (Figure 2D). It is presumed that the presence of Phe affects the agglomeration of the PS-NP, allowing it to easily pass through the chorion of the zebrafish embryos. These results are consistent with those of the particle size analysis, where there were decreases in the particle sizes of PS-NP + Phe, compared to those of the PS-NP (Table S1). After hatching, fluorescence was mainly detected in the yolk of the larvae in all experimental groups. In the case of the PS-NP + Phe experimental group, fluorescence was also detected in some heads where the Phe concentration was higher (Figure 2B). This presumably occurred because when zebrafish embryos are exposed to PS-NP, it initially accumulates mainly in the lipid-rich areas, such as the yolk, and it then moves to the heart and brain through blood vessels and the digestive system [2].

#### 3.3.2. Effect of Mu on the Absorption and Toxicity of PS-NP, Phe and PS-NP + Phe 

Based on the pre-test for selecting the exposure concentration of Mu, 50 μg/mL was selected, as it showed the highest hatching rate and a decreasing abnormality rate; furthermore, 5.0 mg/L of PS-NP and 1.0 mg/L Phe was selected, since they showed the highest toxic effects in the toxicity test.

In the experimental group exposed to PS-NP and PS-NP + Phe, it was observed that the PS-NP was agglomerated and attached to the chorion of the zebrafish embryos (Figure S3b,d). However, in the experimental group with PS-NP + Mu and PS-NP + Phe + Mu, the chorion was clean without the adsorption of anything (Figure S3f,h). This result confirms that Mu can adsorb and reduce PS-NP concentration. 

**Figure 3:**
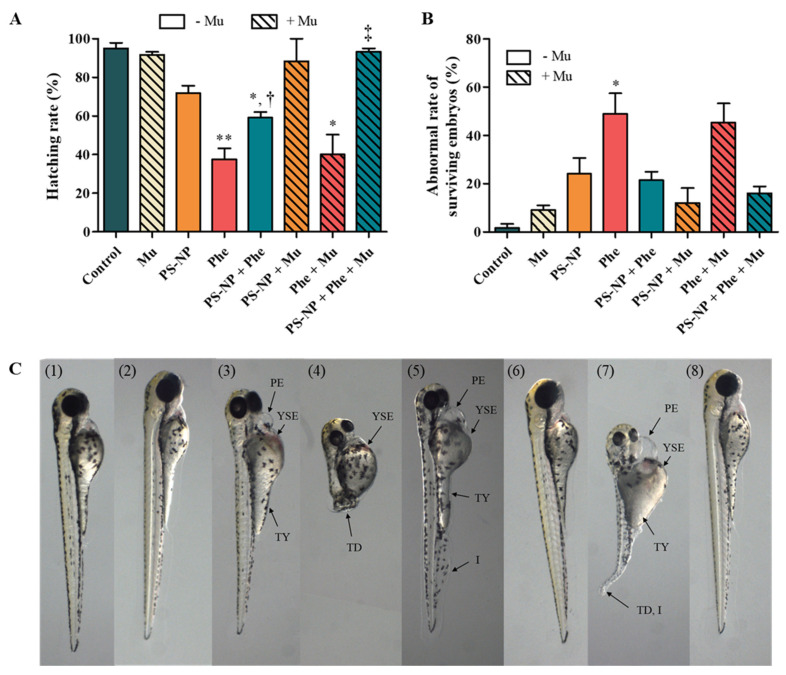
(**A**) Hatching rate and (**B**) abnormal rate of zebrafish. *: control vs. experimental group; (*p* < 0.05). **: control vs. experimental group; (*p* < 0.01), ^†^: Phe vs. PS-NP + Phe; (*p* < 0.05), ^‡^: PS-NP + Phe vs. PS-NP + Phe + Mu; (*p* < 0.05). (**C**) Condition of zebrafish larva exposed to (**1**) control, (**2**) Mu (50 μg/mL), (**3**) PS-NP (5.0 mg/L), (**4**) Phe (1.0 mg/L), (**5**) PS-NP + Phe, (**6**) PS-NP + Mu, (**7**) Phe + Mu, and (**8**) PS-NP + Phe + Mu (PE, pericardial edema; YSE, yolk-sac edema; TY, thicker yolk; TD, tail deformity; I, inflammation).

To confirm that the binding of Mu and PS-NP reduces toxicity in the aquatic ecosystem exposed to PS-NP, zebrafish embryos were exposed and checked for hatching and abnormality rates. The hatching and abnormality rates for the Mu-exposed group were 91.67 ± 2.89% (Figure 3A) and 9.00 ± 3.46% (Figure 3B), respectively, which were not statistically different from the control group. Additionally, the experimental group that was exposed to PS-NP + Mu and PS-NP + Phe + Mu did not show any morphological abnormalities, compared to the control group (Figure 3C).

For up to 48–72 h, the chorion surrounding the zebrafish embryo plays an important role in transporting oxygen, nutrients, and excreta [69]. The diameter of the pores on the chorion was 500–700 nm, and based on the particle size analysis result of PS-NP + Mu (3392 ± 129.90 nm) and PS-NP + Phe + Mu (7277 ± 277.3 nm), the particles could not pass through (Table 2). Therefore, it was inferred that toxicity could be reduced for the experimental group that included Mu because the PS-NP could not penetrate through the chorion of the zebrafish embryo. Additionally, considering its size, the PS-NP can penetrate cell membranes through endocytosis (such as phagocytosis and pinocytosis) and passive transport, leading to the penetration of various biological structures [70]. Thus, an increase in particle size reduces the toxicity, by hampering their permeation through the cell membrane. Therefore, the hatching rate of PS-NP + Mu increased by 16.50 ± 6.85%, compared to that of the PS-NP (Figure 3A). The hatching rate of PS-NP + Phe + Mu also increased significantly by 34.22 ± 5.96%, compared to that of PS-NP + Phe (*p* < 0.05) (Figure 3A). These results were also confirmed by the abnormality rate, which decreased by 12.08 ± 12.10% and 5.44 ± 5.56% for PS-NP + Mu and PS-NP + Phe + Mu, respectively, compared to that of the PS-NP and PS-NP + Phe (Figure 3B). This further confirms that toxicity can be reduced by the addition of Mu because it combines with the PS-NP to prevent absorption into the zebrafish embryo. This is consistent with the results of a previous study that the presence of protein inhibits the attachment of silica nanoparticles (SNPs) to the cell surface, by forming protein corona with the SNPs and reduces the efficiency of absorption into the cell, thus, reducing toxicity [71].

The hatching and abnormality rates for Phe + Mu group was 40.00 ± 18.03% and 45.33 ± 13.65%, respectively, which were comparatively slightly higher or similar to that of Phe (37.50 ± 19.83% and 48.92 ± 29.42%, respectively) (Figure 3A,B). Additionally, Phe + Mu exhibited greater complex morphological abnormalities, such as tail deformity, thicker yolk, and bleeding than the other experimental groups that included Mu (Table 4). This is presumably because of the binding characteristics of Mu and PS-NP. Mu has a flexible string structure composed of alternating hydrophilic parts and pure hydrophobic protein, because of which nanoparticles can be confined using low-affinity bonds that form and decompose quickly and easily [41]. Due to these characteristics, it is presumed that toxicity is not reduced, because Phe is not a particle and cannot effectively bind Mu.

**Figure 4:**
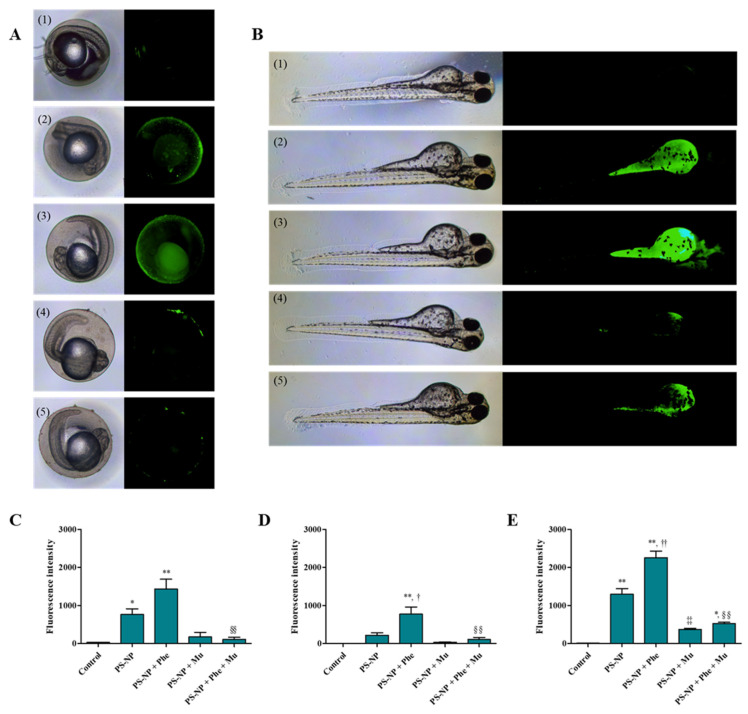
(**A**) Accumulation of PS-NP, according to Phe and Mu in zebrafish embryo and (**B**) larva. (**1**) Control, (**2**) PS-NP, (**3**) PS-NP + Phe, (**4**) PS-NP + Mu and (**5**) PS-NP + Phe + Mu. (**C**) Quantitative analysis of the fluorescence intensity of the zebrafish embryo 24 h after exposure, (**D**) yolk of the zebrafish embryo 24 h after exposure and (**E**) zebrafish larva 72 h after exposure. *: control vs. experimental group; (*p* < 0.05), **: control vs. experimental group; (*p* < 0.01), ^†^: PS-NP vs. PS-NP + Phe; (*p* < 0.05), ^††^: PS-NP vs. PS-NP + Phe; (*p* < 0.01), ^‡‡^: PS-NP vs. PS-NP + Mu; (*p* < 0.01), ^§§^: PS-NP + Phe vs. PS-NP + Phe + Mu; (*p* < 0.01).

The binding between Mu and the PS-NP not only increases the particle size, but also prevents the PS-NP from directly interacting with the outer cell membrane [72]. Fluorescence was detected in the zebrafish yolk of the PS-NP and PS-NP + Phe experimental groups 24 h after exposure, whereas it was not detected in the PS-NP + Mu and PS-NP + Phe + Mu experimental groups (Figure 4A). The fluorescence intensity of the experimental group exposed to PS-NP + Mu decreased by 7.00-fold, compared to that of the PS-NP group, and it was significantly reduced by 7.70-fold (*p* < 0.01) in the PS-NP + Phe + Mu group, compared to that of the PS-NP + Phe group (Figure 4D). Thus, it is inferred that Mu masked the PS-NP surface to prevent it from permeating through the chorion of the zebrafish embryos. An identical pattern was observed after 72 h of exposure. Smaller amounts of fluorescence were detected in the yolk of the PS-NP + Mu and PS-NP + Phe + Mu experimental groups, than in those of the PS-NP and PS-NP + Phe groups (Figure 4B). In the experimental group exposed to PS-NP + Mu, the fluorescence intensity was significantly reduced by 3.56-fold, compared to that of the PS-NP group (*p* < 0.01), and for the PS-NP + Phe + Mu group, it was significantly reduced by 4.30-fold, compared to that of PS-NP + Phe group (*p* < 0.01) (Figure 4E). Some fluorescence was observed in the yolk of the zebrafish in the experimental group exposed to PS-NP + Mu and PS-NP + Phe + Mu, which was presumably due to the removal of the chorion that protects the larvae for up to 48–72 h; some PS-NPs that were not attached to Mu were directly absorbed through the oral and dermal pathways [73]. The experimental group exposed to PS-NP + Phe showed more fluorescence than the PS-NP group (Figure 4A,B), which was consistent with the results presented in Figure 2. 

### 3.4. Gene Analysis

**Figure 5:**
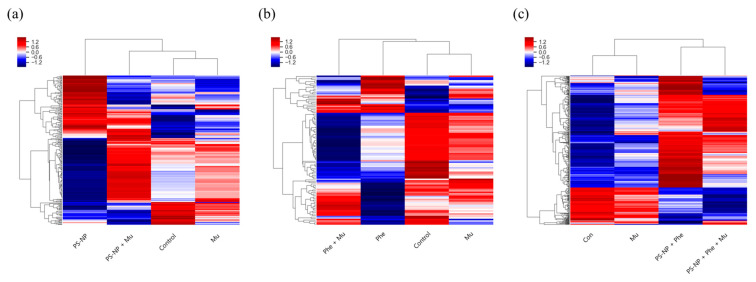
Hierarchical clustering analysis. Differentially expressed genes of (**a**) PS-NP, (**b**) Phe, and (**c**) PS-NP + Phe groups.

Among the 14,435 genes expressed through mRNA sequencing, 246, 104 and 550 genes expressed differently in each experimental group exposed to PS-NP, Phe, and PS-NP + Phe, respectively, compared with the experimental group exposed to Mu; the results were presented in a clustering heatmap (Figure 5). In all three comparisons, the gene expression of the Mu-exposed group was the most similar to that of the control group. The group exposed to PS-NP + Mu showed a similar expression pattern as the control group, and the groups exposed to PS-NP and PS-NP + Mu were clearly opposite in the increase and decrease pattern of gene expression (Figure 5a). However, the group exposed to Phe + Mu exhibited greater differences in the positions of the expressed genes than the Phe-exposed group, and it showed the lowest similarity to the control group in the expression patterns (Figure 5b). Furthermore, the expression patterns of the groups exposed to PS-NP + Phe and PS-NP + Phe + Mu showed a high degree of similarity and were distinctly different from those of the control group (Figure 5c).

**Figure 6:**
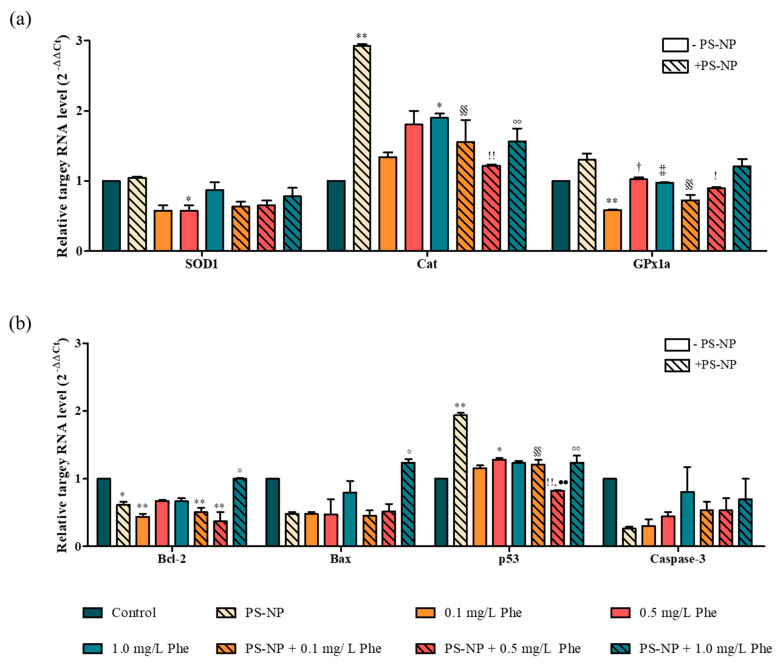
Relative target RNA level (2^−^^ΔΔCt^). (**a**) Genes related to oxidative stress and (**b**) apoptosis. *: control vs. experimental group; (*p* < 0.05), **: control vs. experimental group; (*p* < 0.01), ^†^: 1.0 mg/L Phe vs. 0.5 mg/L Phe; (*p* < 0.05), ^‡‡^: 1.0 mg/L Phe vs. 1.0 mg/L Phe; (*p* < 0.01), ^§§^: PS-NP vs. PS-NP + 0.1 mg/L Phe; (*p* < 0.01), !: PS-NP vs. PS-NP + 0.5 mg/L Phe; (*p* < 0.05), ^!!^: PS-NP vs. PS-NP + 0.5 mg/L Phe; (*p* < 0.01), ^◦^: PS-NP vs. PS-NP + 1.0 mg/L Phe; (*p* < 0.05), ^◦◦^: PS-NP vs. PS-NP + 1.0 mg/L Phe; (*p* < 0.01), ^••^: 0.5 mg/L Phe vs. PS-NP + 0.5 mg/L Phe; (*p* < 0.01).

SOD1 and CAT are the most widely used indicators for monitoring oxidative stress, and they decompose ROS into hydrogen peroxide and oxygen to protect cells and tissues from damage caused by oxidative stress [74]. According to the qPCR analysis result, in the experimental group exposed to PS-NP, the gene expression level of CAT was significantly upregulated (*p* < 0.01) (Figure 6a). This is presumably the activity of CAT for the removal of excessive ROS, generated by the exposure of the PS-NP. The activation of the p53 gene, in response to DNA damage, may lead to the induction of apoptosis [75]. In contrast, the Bcl-2 gene suppresses apoptosis, and its expression can induce apoptosis regardless of the expression of Bax, a gene that promotes apoptosis [76]. In addition, caspase-3 is involved in the activity of caspase, which breaks down cellular proteins and is directly related to apoptosis [77]. The experimental group exposed to PS-NP was not significantly different from the control group in the gene expression of caspase-3 and Bax, but the gene expression of p53 was significantly upregulated (*p* < 0.01) and the Bcl-2 gene was significantly downregulated (*p* < 0.05) (Figure 6b). This indicates that exposure to PS-NP affected the induction of zebrafish apoptosis. In the zebrafish embryogenesis test, conducted earlier for the Phe experimental group, the toxicity increased in a dose-dependent manner, and it was presumed that the expression of the related toxic genes would also change dose-dependently. However, the SOD1 and p53 genes were significantly downregulated or upregulated, respectively, only in the 0.5 mg/L Phe experimental group (*p* < 0.05). GPx1a and Bcl-2 gene expression showed significant downregulation only in the 0.1 mg/L Phe experimental group (*p* < 0.01). This suggests that Phe does not have a direct impact on the gene pathways that induce oxidative stress and apoptosis, and further studies on the gene expression in other pathways are, therefore, required. Furthermore, the experimental group exposed to PS-NP + Phe was significantly different from the control group in the expression of some genes, but no significant change was observed in the PS-NP + Phe experimental group, compared to the PS-NP and Phe experimental groups. These results were similar to those derived from the zebrafish embryogenesis test.

## 4. Conclusions

The collective effect of the PS-NPs exposed to the aquatic ecosystem was confirmed using Mu extracted from *Aurelia aurita*. The physicochemical characteristics of the PS-NP and Phe before and after binding with Mu were analyzed, and it was confirmed through the zebrafish embryonic development experiment whether the biotoxicity caused by Phe, which binds readily with the PS-NP, could be reduced by Mu. The binding capacity of the fluorescent PS-NP and Mu was evaluated using a fluorescence spectrophotometer. PS-NPs absorbed at each embryonic stage were observed under a fluorescence microscope and evaluated through fluorescence quantification. Furthermore, the genotoxicity of the PS-NP, Phe, and the mixtures of PS-NP and Phe, before and after binding with Mu, were compared through mRNA sequence analysis, and a relative quantitative analysis of the expression of specific genes (SOD1, CAT, GPx1a, Bcl-2, Bax, p53, caspase-3) was performed. The following conclusions can be made:

The PS-NP bonded with Mu to form agglomerates and the size of the PS-NP increased from 50 nm to 3392 ± 129.90 nm after binding to Mu. The particle size of PS-NP + Phe was 1614 ± 85.56 nm, which was less compared to the PS-NP, but increased to 7277 ± 277.3 nm after binding with Mu. Based on the results of the binding capacity evaluation of the PS-NP and Mu, Mu combined with 92.84% of the PS-NP concentration after 0.5 h of injection, and the binding capacity was continuously maintained, even after 8 h. This result indicates that Mu can effectively bind with PS-NP. A zebrafish embryogenesis test was conducted to confirm the reduction in toxicity by Mu, the hatching rate in PS-NP + Mu and that the PS-NP + Phe + Mu groups increased compared to the PS-NP and Phe groups, while the abnormality rate decreased. Additionally, it was observed through the fluorescence microscope that the presence of Mu obstructed the absorption of PS-NP and PS-NP + Phe by the zebrafish. When the PS-NP was exposed to the zebrafish embryos at a concentration of 5.0 mg/L, the hatching rate was significantly different from the control group, and the expression of CAT and p53 significantly upregulated, whereas the expression of Bcl-2 significantly downregulated. Furthermore, the results of mRNA sequencing showed that the gene expression of the test groups containing Mu was similar to that of the control group. In summary, these results imply that Mu can be used as a biological material for binding and removing PS-NPs from aquatic environments, resulting in a reduction in toxicity.

## Figures and Tables

**Table 1:** Primer sequence used in qPCR.

Gene	Sequence (5′→3′)		References
	Forward	Reverse	
β-actin	CGGGTGGAGTTTGACACTT	CTCCCTGATGCTGGGTCGTC	[50]
SOD1	GGCCAACCGATAGTGTTAGA	CCAGCGTTGCCAGTTTTTAG	[51]
CAT	AGGGCAACTGGGATCTTACA	TTTATGGGACCAGACCTTGG	[50]
GPx1a	ACCTGTCCGCGAAACTATTG	TGACTGTTGTGCCTCAAAGC	[50]
Bcl-2	TCACTCGTTCAGACCCTCAT	ACGCTTTCCACGCACAT	[50]
Bax	GGCTATTTCAACCAGGGTCC	TGCGAATCACCAATGCTGT	[50]
p53	GGGCAATCAGCGAGCAAA	ACTGACCTTCCTGAGTCTCCA	[50]
Caspase-3	CCGCTGCCCATCACTA	ATCCTTTCACGACCATCT	[50]

**Table 2:** The binding ability of Mu for the PS-NP after 0.5, 1, 2, 4, and 8 h of exposure. **: Concentration of PS-NP at 0 h vs. 1, 2, 4, and 8 h after exposure; (*p* < 0.01).

Time (h)	PS-NP Concentration (mg/L)
0	2.00 ± 0.00
0.5	0.14 ± 0.04 **
1	0.19 ± 0.01 **
2	0.21 ± 0.03 **
4	0.17 ± 0.03 **
8	0.14 ± 0.01 **

**Table 3:** Specific morphological abnormal rate of zebrafish larvae exposed to PS-NP (5.0 mg/L), Phe (0.1, 0.5, and 1.0 mg/L), and PS-NP (5.0 mg/L) + Phe (0.1, 0.5, and 1.0 mg/L). *: control vs. experimental group; (*p <* 0.05), **: control vs. experimental group; (*p <* 0.01), ^††^: 0.1 mg/L Phe vs. 1.0 mg/L Phe; (*p <* 0.01), ^‡^: 0.5 mg/L Phe vs. 1.0 mg/L Phe; (*p* < 0.05), ^§^: 1.0 mg/L Phe vs. PS-NP + 1.0 mg/L Phe; (*p <* 0.05).

	Control	PS-NP	0.1 mg/L Phe	0.5 mg/L Phe	1.0 mg/L Phe	PS-NP + 0.1 mg/L Phe	PS-NP + 0.5 mg/L Phe	PS-NP + 1.0 mg/L Phe
Inflammation	2.50 ± 3.12	13.11 ± 15.16	3.44 ± 8.05	10.44 ± 7.83	32.67 ± 30.00 **^,^^††, ‡^	0.00 ± 0.00	0.00 ± 0.00	7.67 ± 4.03 ^§^
Tail deformity	1.33 ± 3.67	0.00 ± 0.00	0.00 ± 0.00	0.89 ± 2.67	0.00 ± 0.00	1.00 ± 2.45	1.50 ± 3.67	1.33 ± 3.26
Pericardial edema	3.92 ± 4.67	5.56 ± 7.18	3.44 ± 4.48	15.22 ± 7.07 *	9.33 ± 11.88	9.5 ± 10.43	4.33 ± 7.42	8.83 ± 11.03
Yolk-sac edema	3.83 ± 3.71	4.67 ± 5.05	6.22 ± 9.22	10.22 ± 9.92	12.00 ± 10.92	6.83 ± 7.33	8.50 ± 10.77	8.83 ± 11.03
Thicker yolk	0.00 ± 0.00	0.00 ± 0.00	0.78 ± 2.33	0.00 ± 0.00	0.00 ± 0.00	0.00 ± 0.00	0.00 ± 0.00	0.00 ± 0.00
Slight yolk	0.00 ± 0.00	0.00 ± 0.00	0.00 ± 0.00	0.78 ± 2.33	2.44 ± 7.33	1.00 ± 2.45	0.00 ± 0.00	1.33 ± 3.27
Blood vessel	0.00 ± 0.00	6.89 ± 8.43	0.89 ± 2.67	2.89 ± 6.25	12.22 ± 33.08	2.33 ± 3.67	4.00 ± 4.38	1.33 ± 3.27
Blood	2.42 ± 4.72	7.89 ± 7.46	6.00 ± 12.73	10.44 ± 9.41	9.00 ± 9.46	6.83 ± 6.77	7.67 ± 6.71	6.00 ± 7.18
Spinal curvature	1.83 ± 3.59	6.78 ± 10.08	2.67 ± 4.39	4.56 ± 4.61	2.89 ± 4.37	1.67 ± 4.08	2.83 ± 4.40	0.00 ± 0.00

**Table 4:** Specific morphological abnormal rate of fry larva exposed to PS-NP, Phe, PS-NP + Phe, Mu, PS-NP + Mu, Phe + Mu, and PS-NP + Phe + Mu. *: control vs. experimental group; (*p <* 0.05), **: control vs. experimental group; (*p <* 0.01), ^†^: abnormal rate of surviving embryos of Phe vs. PS-NP + Phe; (*p <* 0.05), ^‡^: abnormal rate of surviving embryos of Phe vs. Phe + Mu; (*p <* 0.05), ^‡‡^: abnormal rate of surviving embryos of Phe vs. Phe + Mu; (*p <* 0.01).

	Control	Mu	PS-NP	Phe	PS-NP + Phe	PS-NP + Mu	Phe + Mu	PS-NP + Phe + Mu
Inflammation	0.00 ± 0.00	2.00 ± 3.46	9.83 ± 14.22	34.25 ± 25.85 *	7.67 ± 3.20 ^†^	12.00 ± 10.82	35.33 ± 22.19	7.33 ± 6.35
Tail deformity	0.00 ± 0.00	0.00 ± 0.00	0.00 ± 0.00	2.58 ± 6.33	0.89 ± 2.67	0.00 ± 0.00	11.33 ± 10.26 *^,^^‡^	0.00 ± 0.00
Pericardial edema	1.67 ± 2.89	1.67 ± 2.89	4.17 ± 6.62	11.42 ± 11.10	5.89 ± 9.78	2.67 ± 4.62	22.00 ± 6.25	3.67 ± 6.35
Yolk-sac edema	0.00 ± 0.00	2.00 ± 3.46	4.83 ± 5.84	21.58 ± 20.65	8.56 ± 9.72	0.00 ± 0.00	24.67 ± 4.51	3.67 ± 6.35
Thicker yolk	0.00 ± 0.00	0.00 ± 0.00	0.42 ± 1.44	0.00 ± 0.00	0.00 ± 0.00	2.67 ± 4.62	11.33 ± 10.26 **^, ‡‡^	3.33 ± 2.89
Slight yolk	0.00 ± 0.00	2.00 ± 3.46	0.00 ± 0.00	6.25 ± 9.63	0.89 ± 2.67	0.00 ± 0.00	4.67 ± 8.08	0.00 ± 0.00
Blood vessel	0.00 ± 0.00	2.00 ± 3.46	6.58 ± 7.22	12.50 ± 29.89	5.00 ± 7.14	0.00 ± 0.00	0.00 ± 0.00	0.00 ± 0.00
Blood	0.00 ± 0.00	0.00 ± 0.00	6.92 ± 7.23	10.42 ± 10.05	5.67 ± 6.10	0.00 ± 0.00	24.67 ± 4.51 **	3.67 ± 6.35
Spinal curvature	0.00 ± 0.00	0.00 ± 0.00	5.08 ± 9.13	3.08 ± 4.62	1.67 ± 5.00	1.67 ± 2.89	0.00 ± 0.00	3.33 ± 2.89

## Data Availability

The data presented in this study are available on request from the corresponding author.

## Supplementary Materials

The following are available online at https://www.mdpi.com/article/10.3390/nano12091427/s1, Table S1. Particle diameter and zeta potential analyses of PS-NP, PS-NP + Mu, PS-NP + Phe, and PS-NP + Phe + Mu. Figure S1. (a) Fluorescence intensity by concentration of PS-NP, (b) standard curve generated from the obtained fluorescence intensity. Linear relationship between fluorescence intensity and concentration of PS-NP. Figure S2. FE-SEM images of (a) PS-NP, (b) PS-NP + Phe, (c) Mu, (d) PS-NP + Mu, and (e) PS-NP + Phe + Mu. Figure S3. (a) Control, (b) PS-NP (5.0 mg/L), (c) Phe (1.0 mg/L), (d) PS-NP + Phe, (e) Mu (50 μg/mL), (f) PS-NP + Mu, (g) Phe + Mu, and (h) PS-NP + Phe + Mu after exposure of 32 h (arrow in (b) represents PS-NP, and arrow in (d) represents PS-NP + Phe).
