# Supplementary material for: Reduction in Toxicity of Polystyrene Nanoplastics Combined with Phenanthrene through Binding of Jellyfish Mucin with Nanoplastics
Source: Nanomaterials (Basel). 2022 Apr 22;12(9):1427. doi: 10.3390/nano12091427 (PMC9105387; doi:10.3390/nano12091427)
Supplement: Supplementary file 1 [file nanomaterials-12-01427-s001.zip › nanomaterials-1653031-supplementary.pdf]

nanomaterials

Supplementary Materials

Sun Woo Geum [1] and Min-Kyeong Yeo [1,2,*]

1 Department of Applied Environmental Science, Graduate School of Engineering, Kyung Hee University, 1732 Deogyeong-daero, Giheung-gu, Yongin-si, Gyeonggi-do 17104, Republic of Korea
2 Department of Environmental Science and Engineering, College of Engineering, Kyung Hee University, 1732 Deogyeong-daero, Giheung-gu, Yongin-si, Gyeonggi-do 17104, Republic of Korea
* Corresponding author's e-mail addresses and phone numbers:
Min-Kyeong Yeo: bioclass@khu.ac.kr; +82-31-201-2413

Table S1. Particle diameter and zeta potential analyses of PS-NP, PS-NP + Mu, PS-NP + Phe, and PS-NP + Phe + Mu.

	Diameter [nm]	Zetapotential [mV]
PS-NP	2132 ± 90.86	-7.49 ± 1.12
PS-NP + Mu	3392 ± 129.90	-3.37 ± 0.58
PS-NP + Phe	1614 ± 85.56	-12.34 ± 1.95
PS-NP + Phe + Mu	7277 ± 277.3	-5.13 ± 0.07

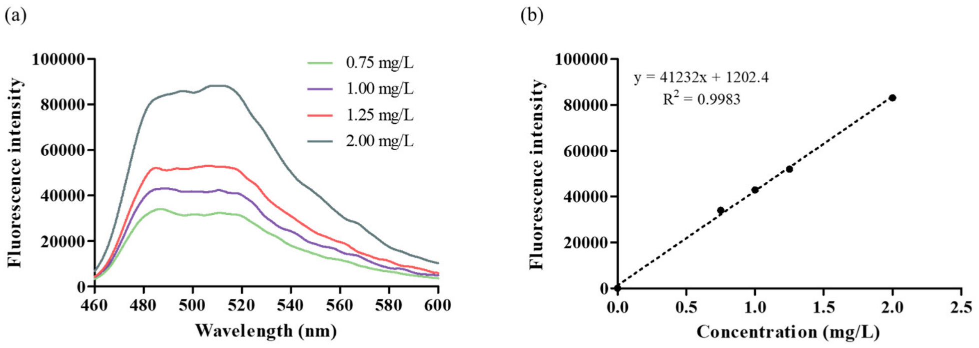

Figure S1. (a) Fluorescence intensity by concentration of PS-NP, (b) standard curve generated from the obtained fluorescence intensity. Linear relationship between fluorescence intensity and concentration of PS-NP.

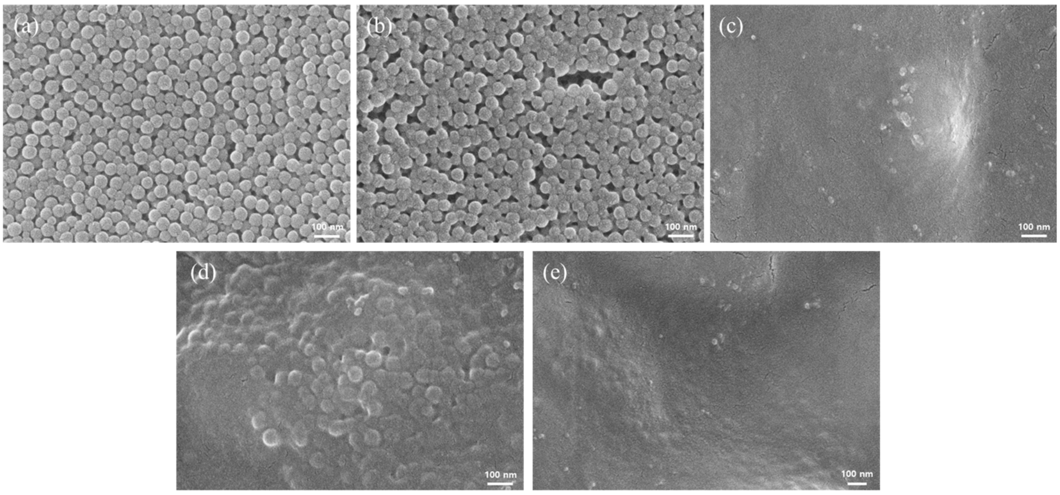

Figure S2. FE-SEM images of (a) PS-NP, (b) PS-NP + Phe, (c) Mu, (d) PS-NP + Mu, and (e) PS-NP + Phe + Mu.

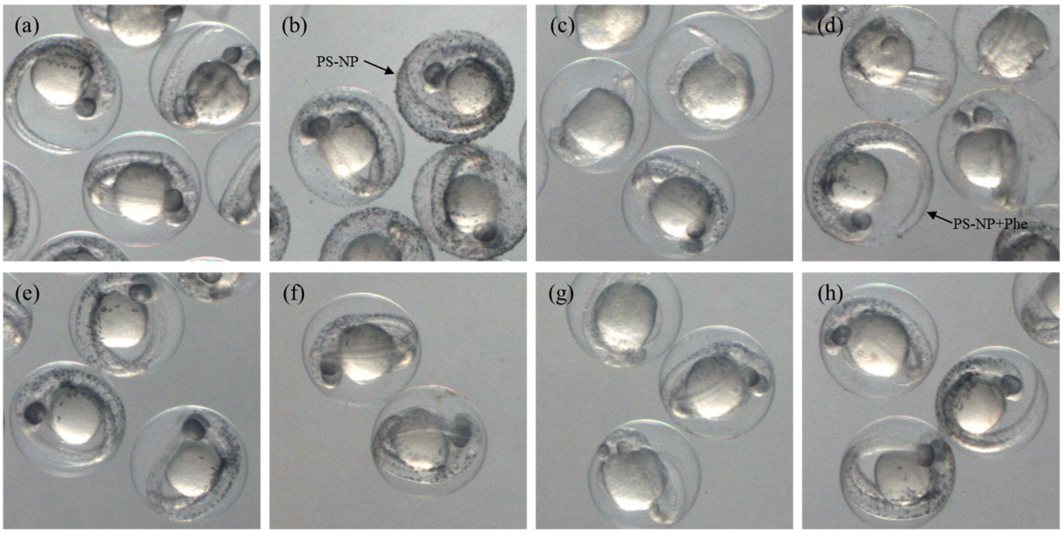

Figure S3. (a) Control, (b) PS-NP (5.0 mg/L), (c) Phe (1.0 mg/L), (d) PS-NP + Phe, (e) Mu (50 μg/mL), (f) PS-NP + Mu, (g) Phe + Mu, and (h) PS-NP + Phe + Mu after exposure of 32 h; (arrow in (b) represents PS-NP, and arrow in (d) represents PS-NP + Phe).
